# An Immunosuppressive Tick Salivary Gland Protein DsCystatin Interferes With Toll-Like Receptor Signaling by Downregulating TRAF6

**DOI:** 10.3389/fimmu.2018.01245

**Published:** 2018-06-01

**Authors:** Ta Sun, Fanqi Wang, Wen Pan, Qihan Wu, Jingwen Wang, Jianfeng Dai

**Affiliations:** ^1^Institutes of Biology and Medical Sciences, Jiangsu Key Laboratory of Infection and Immunity, Soochow University, Suzhou, China; ^2^Key Laboratory of Reproduction Regulation of NPFPC, SIPPR, RID, Fudan Unversity, Shanghai, China; ^3^School of Life Science, Fudan University, Shanghai, China

**Keywords:** tick, cystatin, immunosuppressant, TLR signaling, inflammation

## Abstract

Ticks, blood-feeding arthropods, and secrete immunosuppressive molecules that inhibit host immune responses and provide survival advantages to pathogens. In this study, we characterized the immunosuppressive function of a novel tick salivary protein, DsCystatin, from *Dermacentor silvarum* of China. DsCystatin directly interacted with human Cathepsins L and B and inhibited their enzymatic activities. DsCystatin impaired the expression of inflammatory cytokines such as IL1β, IFNγ, TNFα, and IL6 from mouse bone marrow-derived macrophages (BMDMs) that had been stimulated with LPS or *Borrelia burgdorferi*. Consistently, DsCystatin inhibited the activation of mouse BMDMs and bone marrow-derived dendritic cells by downregulating the surface expression of CD80 and CD86. Mechanically, DsCystatin inhibited LPS- or *B. burgdorferi*-induced NFκB activation. For the first time, we identified that DsCystatin-attenuated TLR4 signaling by targeting TRAF6. DsCystatin enhanced LPS-induced autophagy, mediated TRAF6 degradation *via* an autophagy dependent manner, thereby impeded the downstream phosphorylation of IκBα and the nuclear transport of NFκB. Finally, DsCystatin relieved the joint inflammation in *B. burgdorferi* or complete Freund’s adjuvant induced mouse arthritis models. These data suggested that DsCystatin is a novel immunosuppressive protein and can potentially be used in the treatment of inflammatory diseases.

## Introduction

Ticks are blood-feeding arthropods that can be temporarily parasitic on the surface of vertebrate. During feeding, ticks transmit various pathogens that cause human diseases including Lyme disease, tick-borne encephalitis, and human granulocytic anaplasmosis, among others (1, 2). Unlike other vectors that feed in a very rapid process, ticks spend a relatively long period of time feeding on animals. Soft ticks feeding lasts several hours, whereas hard ticks feeding lasts for 6–9 days (1). Tick saliva contains a large number of biological active molecules, which interfere with host defense response and benefit for tick feeding and pathogen transmission (3, 4).

Mammalian Cathepsin family is a class of proteases found in cells (especially in lysosomes) of various animal tissues. It consists of Cathepsins A, B, C, D, E, L, S, and other enzymes (5). In immune cells, Cathepsins are involved in antigen degradation, procession and presentation, and are key enzymes that initiate the immune response (5, 6). For example, Cathepsins mediate proteolytic cleavage of TLRs (e.g., TLR7 and TLR9), which is a prerequisite for signal transduction (7). Cathepsins also affect the recruitment of MyD88, which is responsible for activation of TLR signaling pathways (8). In addition, Cathepsins are able to directly activate or inhibit certain cytokines, which play an important role in the inflammatory immune response (8). Cathepsin G can process IL-8 precursors to promote IL-8 secretion, and Cathepsin B affects TNFα transport (9). In these physiological processes, Cathepsins are tightly regulated at various levels such as protein expression, proenzymes activation, and regulation by protease inhibitors. Among them, the role of Cathepsin inhibitors is particularly critical (10).

The Cystatin protein family is a natural inhibitor of Cathepsins. Cystatins are widely distributed in various organisms (such as mammals, nematodes, and arthropods) and highly conserved during evolution (10, 11). Cystatins regulate the function of intracellular Cathepsin and maintain the stability of intracellular protein. The Cystatin family includes Cystatin A, B, C, D, E/M, F, G, S, SN, and SA. Cystatin family plays important roles in a lot of cellular events including protein catabolism, inflammation, antigen presentation, and T-cell dependent immune response (11). Cystatin C, the most effective inhibitor of Cathepsin B, H, L, and S, can be transported into the macrophage and dendritic cells (DCs) and modulate their functions (10). Cystatin E/M affects NFκB transport into nucleus and suppresses tumor cell growth (12). Cystatins B and C influence the NO production in macrophages, and this function does not depend on their protease inhibitory activity (13). Cystatin F is highly expressed in CD56^dim^CD16^+^ NK cells and modulates the NK function in sites of inflammation (14).

Tick salivary glands (SG) contain various members of the Cystatin family (15). Tick cystatins belong to class type II Cystatin, which are secreted into the host skin and potentially regulate host immune response (15). Tick cystatin family proteins, such as Sialostatin L, inhibit the activity of host protease in DC and block antigen presentation; impair macrophage and DC activation as well as the inflammatory cytokine secretion (16). In experimental autoimmune encephalomyelitis, arthritis, and asthma mice models, injecting Sialostatin L can significantly attenuate the severity of the diseases (17, 18). Tick Cystatin Sialostatin L2 inhibits caspase-1 activation when the host is infected with *Rickettsia* sp. Sialostatin L2 also binds to Annexin A2 to prevent oligomerization of NLRC4, thereby inhibiting the activation of caspase-1 and the secretion of IL1β and IL-18.

In this study, we characterized a novel Cystatin family protein, DsCystatin, isolated from SG of *Dermacentor silvarum*. *D. silvarum* is a major tick species in northeast China, which can transmit several important human pathogens including *Borrelia burgdorferi* and tick borne encephalitis virus (19). We have found that DsCystatin is an effective anti-inflammatory molecule and can be potentially used in the treatment of inflammatory diseases in mouse models.

## Materials and Methods

### Mice, Ticks, Cell lines, and Spirochetes

Female C3H/HeJ and BALB/c mice (6- to 8-week-old) were purchased from Model Animal Research Center of Nanjing University (Nanjing, China) and maintained in pathogen-free conditions. All animal experiments were performed in accordance with the Guide of National Animal Care and Use committee and the Laboratory Animal Ethical Commission of Soochow University (SYXK2014-0030). *D. silvarum* was collected from forest area of northeast China. RAW264.7, TLR2-HEK293, and TLR4-HEK293 cell lines were obtained from InvivoGen (San Diego, CA, USA) and grown in DMEM supplemented with 10% FBS (Gibco™, #10099141, USA) and antibiotics/antimycotics. *B. burgdorferi* B31 strain (ATCC^®^ 35210™) was grown in Barbour–Stoenner–Kelly-H (Sigma, #B8291, USA) complete medium at 33°C.

### Cloning, Expression, and Purification of Recombinant DsCystatin

Total mRNAs from whole adult *D. silvarum* were prepared and subjected to high-throughput RNA sequencing. The Unigene sequences were obtained and screened for novel Cystatin-like genes by BLAST analyses against the NCBI NR database.^1^

The ORF of DsCystatin was amplified from the cDNA library using the gene-specific primers (Table S1 in Supplementary Material). Then the ORF fragment was cloned into the pGEX-6p2 bacterial expression vector and transformed into *Escherichia coli* strain BL21 (DE3) for protein expression. *E. coli* cultures (OD 0.6–0.8) were stimulated with 0.1 mM isopropyl β-d-thiogalactoside at 30°C for 4 h and GST-tagged DsCystatin was induced as soluble form. Then, the recombinant fusion protein was purified using GST affinity agarose (GE Healthcare, Sweden) and eluted with reduced glutathione (10 mM) (Sigma, USA). The GST tag was removed by Prescission Protease (Sigma, # SAE0045, USA). LPS contamination in purified recombinant proteins was removed by endotoxin-adsorbing medium (Thermo, #20339, USA).

### Generation of Bone Marrow-Derived Dendritic Cells (BMDCs) and Bone Marrow-Derived Macrophages (BMDMs)

Mouse BMDCs and BMDMs were generated as described previously (20, 21). Briefly, mice were sacrificed by cervical dislocation. Intact femurs and tibias were removed, and bone marrow was harvested by repeated flushing with RPMI media supplemented with 10% FCS. After 7 days of culture with GM-CSF (20 ng/ml, PeproTech, #315-03, USA), bone marrow cells were differentiated into DCs. For the generation of BMDM, bone marrow cells were obtained in the same way and induced with RPMI (Hyclone, #AAL210465, USA) + 10% FBS + 10% L929 cell-conditioned medium for 7 days.

### Protease Inhibition Assays

The inhibitory effect of recombinant DsCystatin protein on human Cathepsin L and B was detected by InnoZyme™ Cathepsin Activity Fluorescence Assay Kit (Millipore, #CBA023/CBA001, Germany) and Cathepsin B Enzyme Activity Kit (Sigma, #SRP0289, USA), respectively. To characterize the inhibition of DsCystatin on cellular Cathepsins, Raw264.7 cells were incubated with recombinant DsCystatin, PBS (negative controls, NC), GST, or another recombinant tick protein control Salp17. After 2 h, the cell lysates were harvested, and the total Cathepsin L activity was detected with the above kits.

### GST Pull Down

The recombinant GST-tagged DsCystatin or GST controls were incubated with GST affinity column (GE Healthcare, #1024800, Sweden), respectively. After 2 h, 293T cell lysates were loaded onto the column and incubated at room temperature for another 2 h. Then, the column was washed by PBS to remove the unbound proteins. The GST-tagged proteins were then eluted with reduced glutathione (10 mM), and the DsCystatin interacting proteins were analyzed by Western blot.

### Quantitative Real-Time PCR (qRT-PCR)

Total RNA was extracted using the total RNA kit (OMEGA, #R6834-02, USA) and reverse-transcribed using the PrimeScriptTM Master Mix kit (TaKaRa, #RR037Q, Japan). qRT-PCR was performed using a SYBRGreen-based method with gene-specific primers. The expression levels of selected genes were normalized to mouse β-actin gene. (Oligo-primer sequences for qRT-PCR are shown in Table S1 in Supplementary Material.)

### Measurement of Cytokine Production by qRT-PCR and Enzyme-Linked Immunosorbent Assay (ELISA)

Freshly isolated BMDMs were seeded on 48-well plates (NEST, #748001, China) for 24 h and then preincubated with DsCystatin (4 µM) or control proteins [GST or recombinant tick protein P11, Salp20, and Salp17 (Figure S1 in Supplementary Material)] (4 µM) for 2 h followed by stimulation with LPS (200 ng/ml, Sigma, USA) or *B. burgdorferi* spirochetes (MOI = 1.0). After 4 h, cells were harvested and used for the detection of IL1β, IL-6, TNFα, and IFNγ mRNA expression with qRT-PCR. Cell-free culture supernatants were harvested at 24 h after stimulation and used for the detection of IL1β and TNFα proteins by ELISA (Biolegend, #432604/430904, USA) according to the manufacturer’s instructions.

### OVA Degradation in DCs

Dendritic cells were prepared as described above and pre-incubated with DsCystatin or GST for 2 h followed by incubation with DQ-OVA (1 µg/ml; Invitrogen, #25-5743-82, England) for 3 h. Cells incubated with medium only were used as controls (NC). After repeatedly washing with PBS + 1% FBS at 4°C, these cells were analyzed for the level of OVA degradation by flow cytometry (FL-1 fluorescence channel).

### Flow Cytometry

Bone marrow-derived macrophages and BMDCs were prepared as described above and pre-incubated with DsCystatin and GST for 2 h. After 12 h of stimulation with LPS (50 ng/ml), cells were washed twice and stained with FITC-labeled CD80 (Biolegend, #104705, USA) and PE-labeled CD86 antibodies (Biolegend, #105007, USA). Data were collected using a FACS Calibur flow cytometer (BD, USA), and mean fluorescence intensity (MFI) was analyzed with FlowJo software.

### Luciferase Reporter Assay

293T cells stably overexpressing TLR2 (TLR2-293) and TLR4 (TLR4-293) were seeded on 96-well plates (NEST, #701101, China) and transfected with NFκB-Luc (Firefly luciferase, experimental reporter, 100 ng/well) and pRL-TK reporter (Renilla luciferase, internal control, 5 ng/well) plasmids (Clontech, USA), respectively. 24 h post-transfection, cells were pre-incubated with DsCystatin (4 µM) or GST (4 µM) for 2 h and then stimulated with LPS (200 ng/ml, Sigma, #L2630, USA) or *B. burgdorferi* spirochetes (MOI = 1.0). The luciferase activity was measured after another 24 h, using a Dual Glow kit according to the manufacturer’s instructions (Promega, #E2920, USA).

To assess the effect of DsCystatin on distinct molecules in TLR4 pathway, TLR4-293 cells were seeded on 96-well plates and transfected with NFκB-Luc, pRL-TK, together with MYD88, TRAF6, NEMO, IKKα, or NFκBp65 plasmids, respectively. After 24 h post-transfection, cells were incubated with DsCystatin (4 µM) and GST (4 µM) for 24 h. The activation of NFκB induced by overexpression of distinct signal molecule was detected by luciferase assay as described above.

### Western Blot Analysis

Raw264.7 or TLR4-293 cells were seeded on six-well plates (NEST, #703001, China) and pre-incubated with DsCystatin (4 µM) or GST (4 µM) for 2 h. After stimulation with LPS (200 ng/ml) for 12 h, cell lysates were fractionated by SDS-PAGE and transferred to a polyvinylidene difluoride membrane. The expressions of proteins of TLR4 signaling pathway were analyzed by immunoblot with anti-NFκB p65 (CST, #8242, USA)/anti-p-NFκB p65 (CST, #3033, USA), anti-IκBα (CST, #4814, USA)/anti-p-IκBα (CST, #2859, USA), anti-TRAF6 (CST, #8028, USA), and anti-TAK1 (CST, #5206, USA) antibodies. GAPDH (Abcam, #ab8245, England) and Laminb1 (Proteintech, #12987-1-AP, USA) were served as internal controls. For analysis of endogenous LC3-II, TLR4-293 cells were seeded on 12-well plates and pre-incubated with DsCystatin (4 µM) or GST (4 µM) for 2 h followed by stimulation with LPS (1 µg/ml or 5 µg/ml) for 12 h. (Since TLR4-293 cells are not as sensitive as BMDMs to LPS stimulation, a higher concentration of LPS and a longer incubation period were used here.) Autophagy was analyzed by immunoblotting with LC3-II (CST, #12741, USA) and p62 (MBL, #PM045, Japan) antibodies. An autophagy-specific inhibitor chloroquine (CQ) (0.05 nM, Selleckchem, #S4157, USA) was used to inhibit autophagy during LPS stimulation.

### Fluorescence Microscopy

To investigate NFκB translocation during LPS stimulation, TLR4-293 cells were transfected with NFκBp65 plasmids using Lipofectamine 2000 (Invitrogen, #11668030, USA). At 24 h post-transfection, cells were incubated with DsCystatin (4 µM) or GST (4 µM) for 2 h, followed by stimulation with LPS (200 ng/ml) for 4 h. Cells were stained with anti-p65 antibody (CST, USA), followed by incubation with FITC-labeled anti-mouse IgG (Jackson ImmunoResearch, #BA1101, USA). Nuclei were counterstained with 4,6-diamidino-2-phenylindole (Sigma, #D8417, USA). Cells were examined under a fluorescence microscope (Nikon A1, Japan).

To observe autophagy, TLR4-293 cells were transfected with mCherry-LC3 (500 ng/ml) plasmid for 24 h. Cells were pre-incubated with DsCystatin (4 µM) or GST (4 µM) for 2 h, and then stimulated with LPS (1 µg/ml) for 12 h. Cells were analyzed under a fluorescence microscope.

### *B. burgdorferi*-Induced Arthritis Model

*Borrelia burgdorferi*-induced arthritis model was generated as described previously (22–24). Briefly, 10^3^ *B. burgdorferi*, together with 20 μg DsCystatin or PBS, were injected into the mouse footpad (Five mice per group). At days 7, 14, and 21, the size of the mouse ankle joint was measured, and orbital blood samples were used to analyze the *B. burgdorferi*-specific antibody titers by ELISA. At day 21, mice were sacrificed, and the joints were dissected for pathological analysis. Serial sections of the entire joint were stained with hematoxylin and eosin. The knee sand tibiotarsai were scored for arthritis severity on a scale of 0 (negative) to 3 (severe) in a blinded fashion as described previously (23).

### The Complete Freund’s Adjuvant (CFA)-Induced Arthritis Model

The CFA-induced mouse arthritis was induced by intra-articular injection of 20 µl of CFA (1 mg/ml, Sigma, #F5881, USA) (25, 26). NC group mice were received the same volume of PBS as controls. 24 h later, mice were injected intramuscularly with 20 µg DsCystatin or PBS (as controls) every 2 days. Ankle thickness was measured every 2 days. At day 21 following the CFA injections, mice were sacrificed and joints were dissected for pathological analysis.

### Statistical Analysis

Each experiment was performed at least three times and data are shown as mean + SEM. Statistical differences were analyzed by Student’s *t*-test and ANOVA. A value of *p* < 0.05 was considered statistically significant.

## Results

### DsCystatin Binds to Cathepsin L and B and Inhibits Their Protease Activity

**Figure 1:**
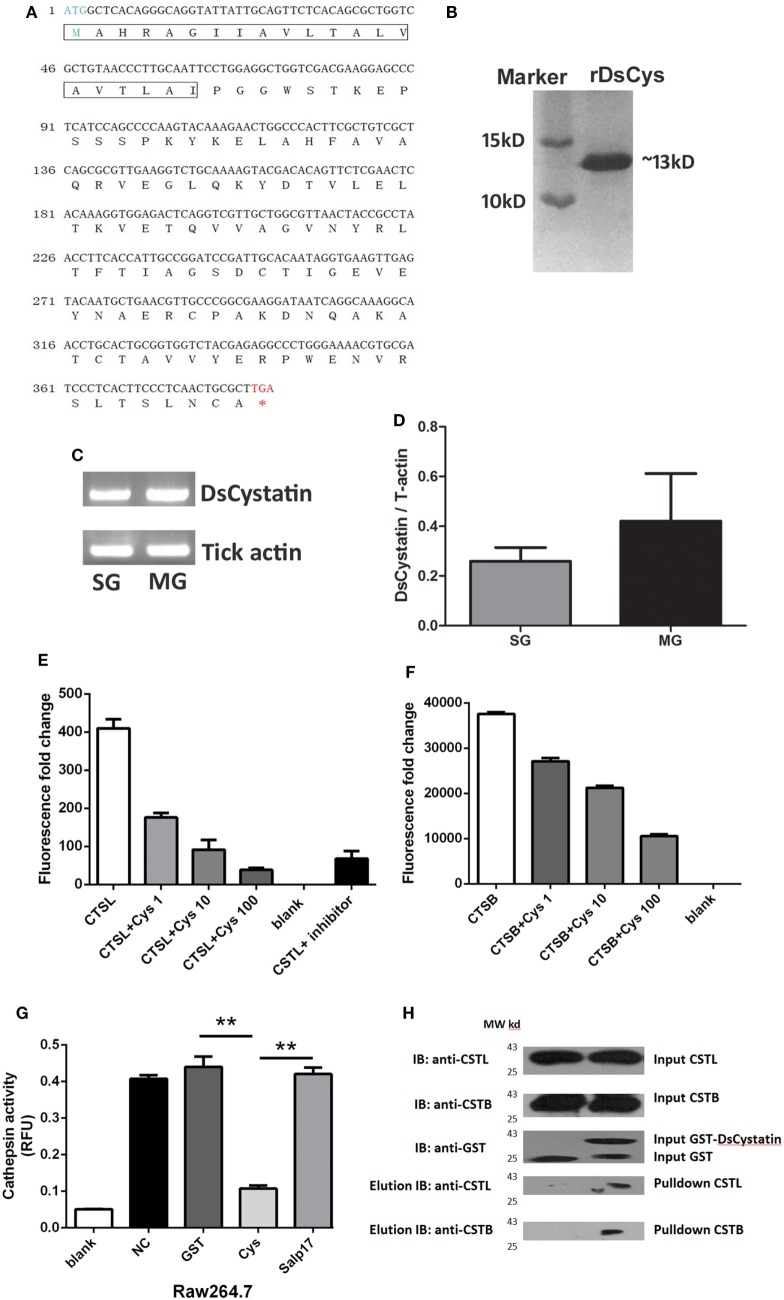
Characterization of DsCystatin and its protease inhibitory activity. **(A)** Nucleotide and deduced amino acid sequence of DsCystatin. The signal peptide sequence is indicated in the frame. **(B)** Recombinant DsCystatin purified from a GST affinity column (after cleavage of the GST tag, SDS-PAGE with Coomassie staining). **(C,D)** Expression of DsCystatin in tick salivary gland (SG) and midgut (MG), **(C)** regular PCR, and **(D)** quantitative real-time PCR. **(E,F)** DsCystatin inhibits Cathepsin L **(E)** and Cathepsin B **(F)** activity in a dose-dependent manner (DsCystatin final concentration: 1, 10, and 100 ng/mL). Blank: no Cathepsin enzymes. **(G)** DsCystatin inhibits Cathepsin L activity in cell lysates from mouse macrophage cell line RAW264.7 cells. [Negative control (NC): PBS control added into the enzyme reaction.] Results are expressed as mean + SEM of three triplicate samples. *p < 0.05 and **p < 0.01 (*t*-test). The representative results from at least three independent experiments are shown. **(H)** DsCystatin binds to human Cathepsin L and B as determined by GST pull-down assay. The representative results from at least three independent experiments are shown.

By sequencing a cDNA library of *D. silvarum*, a novel cDNA clone encoding a putative tick Cystatin gene was obtained and named as DsCystatin (GenBank accession number KJ885300). Sequence analysis indicated that DsCystatin ORF was 387 bp long, encoding a 128-amino acid peptide. The predicted DsCystatin protein shared 90 and 44% of sequence similarity to tick cystatin 2c (AGW80659) and human Cystatin C (NP_000090), respectively. A putative signal peptide cleavage site at amino acid position 21 was also found in DsCystatin (as analyzed by SignalP4.1 software^2^) (Figure 1A). Recombinant DsCystatin was obtained using prokaryotic expression system with molecular weight 12.1 kDa (Figure 1B). Expression of DsCystatin was detected in both tick midgut (MG) and SG with a slightly higher expression in MG (Figures 1C,D).

As Cystatins are natural inhibitors of Cathepsins, we hypothesize that DsCystatin is a potential inhibitor secreted into the mammal during feeding. We tested its inhibitory activity against recombinant human Cathepsin L and B. As shown in Figures 1E,F, DsCystatin inhibited Cathepsin L and Cathepsin B activity in a dose-dependent manner. Cathepsins are abundantly expressed in immune cells, such as Raw264.7 cells, a mouse macrophage cell line. Therefore, we examined the inhibitory effect of DsCystatin on Cathepsins in Raw264.7 cell lysates. Similar to the data in Figures 1E,F, DsCystatin had apparent inhibitory effect on Cathepsins in cell lysates (Figure 1G). Next, we tested whether the inhibition activity was mediated by its direct interaction with Cathepsins. A GST pull-down assay was performed using 293T cell lysates and recombinant GST or GST-DsCystatin proteins. As shown in Figure 1H, DsCystatin pulled out Cathepsin L and B, while GST control protein did not. These results indicated that DsCystatin inhibits Cathepsin L and B activity by direct binding to these host proteases.

### DsCystatin Shows Immunosuppressive Activity on Mouse BMDMs and BMDCs

**Figure 2:**
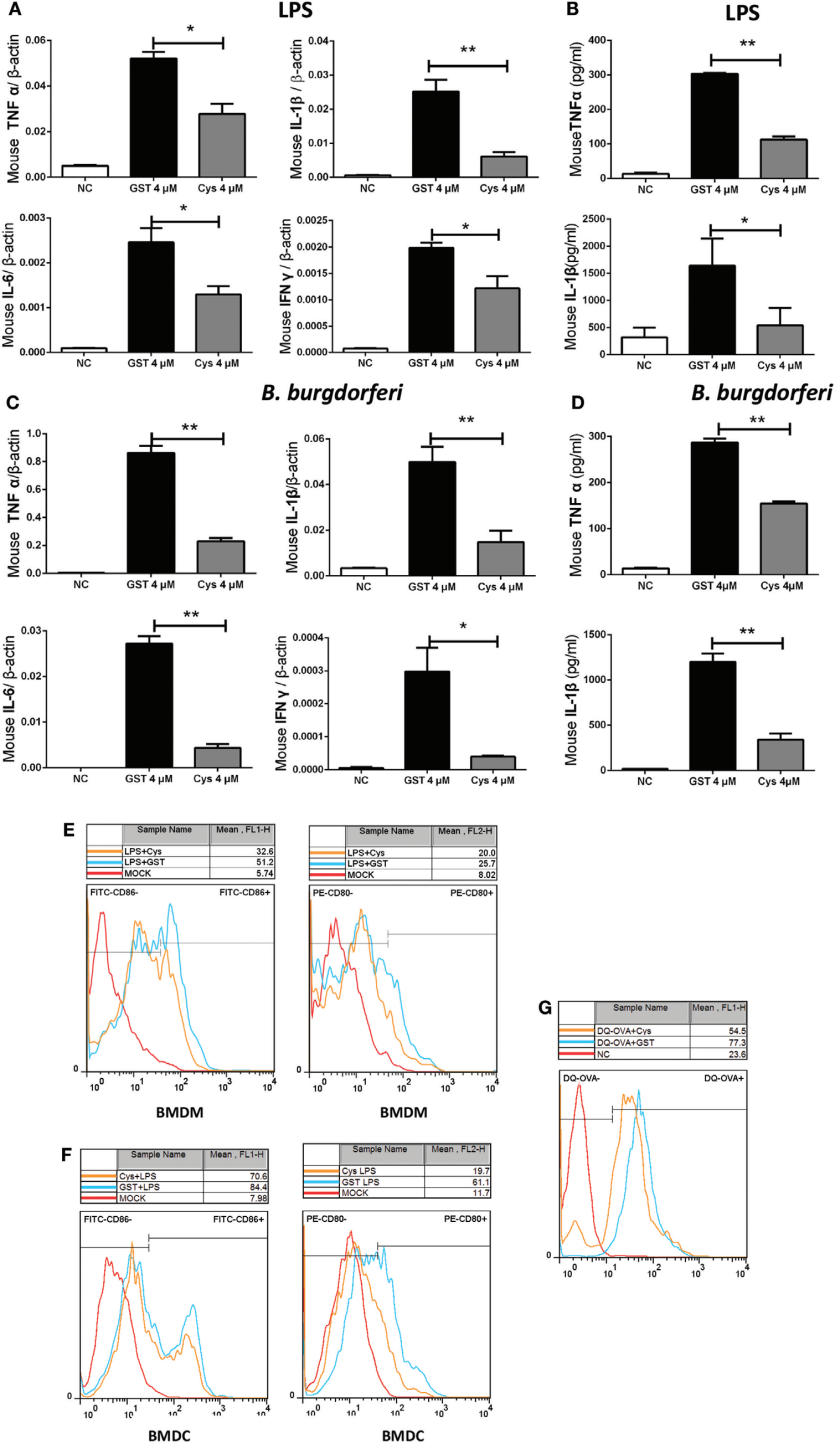
DsCystatin inhibits LPS or *Borrelia Burgdorferi*-stimulated activation of mouse bone marrow-derived macrophages (BMDMs) and bone marrow-derived dendritic cells (BMDCs). **(A,B)** DsCystatin inhibits LPS-induced cytokine production: **(A)** cytokine mRNA level as determined by quantitative real-time PCR and normalized with mouse β-actin gene. **(B)** Cytokine protein level as determined by enzyme-linked immunosorbent assay. **(C,D)** DsCystatin inhibits *B. burgdorferi*-induced cytokine production at mRNA **(C)** and protein **(D)** levels. Cells that were not stimulated with LPS or *B. burgdorferi* were served as negative controls (NC). Results are expressed as mean + SEM of three triplicate samples. *p < 0.05 and **p < 0.01 (*t*-test). The representative results from at least three independent experiments are shown. **(E,F)** DsCystatin downregulates the surface expression of CD80 and CD86 on LPS stimulated BMDMs **(E)** and BMDCs **(F)**. **(G)** DsCystatin inhibits OVA cleavage in BMDCs. Cells were pre-incubated for 3 h at 37°C in the presence or absence of 4 µM DsCystatin and further incubated with DQ-OVA (1 µg/ml) for 2 h at 37°C. OVA fragmentation was analyzed by flow cytometry. The representative results from three independent experiments are shown.

Cytokines produced by activated BMDMs play a key role in the immune response toward external stimulation. We first tested whether the mouse BMDMs response to microbial stimulation is affected by the presence of DsCystatin. Transcriptional level of LPS-induced production of TNFα, IL-6, IL1β, and IFNγ by BMDMs was inhibited by DsCystatin (Figure 2A) [0.25, 1, and 4 µM of DsCystatin were tested for the suppressive effect on cytokine expression, and DsCystatin displays a most dramatic inhibition at 4 µM (Figure S1 in Supplementary Material). For consistency, 4 µM of DsCystatin was used for the following experiments below.] Similar inhibitory effect of DsCystatin on the production of TNFα and IL1β protein was also observed (Figure 2B).

*Borrelia burgdorferi* is a common human pathogen that is carried by *D. silvarum*. *B. burgdorferi* does not express LPS and mainly activates the TLR2 signal pathway (27). We tested whether *B. burgdorferi*-stimulated pro-inflammatory cytokine expression was also affected by DsCystatin. As shown in Figures 2C,D, DsCystatin also inhibited the production of TNFα/IL-6/IL1β/IFNγ transcripts and TNFα/IL1β proteins in *B. burgdorferi*-stimulated BMDMs.

A lot of the research studies have suggested that tick salivary proteins inhibit immune cell activation and maturation (3, 4). We next tested whether the expression of costimulatory molecules in LPS-stimulated mouse immune cells was affected by DsCystatin. Pre-incubation of BMDMs with DsCystatin reduced the expression of CD80 [MFI: 37.1 ± 3.6 (Cystatin) vs 58.8 ± 2.6 (GST), *p* < 0.01] or CD86 [MFI: 26.7 ± 1.5 (Cystatin) vs 37.1 ± 3.6 (GST), *p* < 0.01] induced by LPS when compared with control cells (incubated with GST protein control) (Figure 2E). Similarly, LPS-induced CD80 [MFI: 64.4 ± 3.7 (Cystatin) vs 82.3 ± 1.3 (GST), *p* < 0.05] or CD86 [MFI: 23.9 ± 3.4 (Cystatin) vs 60.1 ± 4.7 (GST), *p* < 0.01] expressions were suppressed by pre-incubation with DsCystatin in BMDCs (Figure 2F). Furthermore, we investigated the effects of DsCystatin on antigen degradation and cleavage. BMDCs were pre-incubated with GST or DsCystatin and pulsed with DQ-OVA, which emits fluorescence upon proteolysis. As shown in Figure 2G, DQ-OVA fluorescence in DCs is reduced in the presence of DsCystatin (MFI: 57.1 ± 0.8 (Cystatin) vs 78.8 ± 2.4 (GST), *p* < 0.01), suggesting that DsCystatin impaired the antigen processing in DCs.

### DsCystatin Inhibits TLR2 and TLR4-Directed NFκB Activation

**Figure 3:**
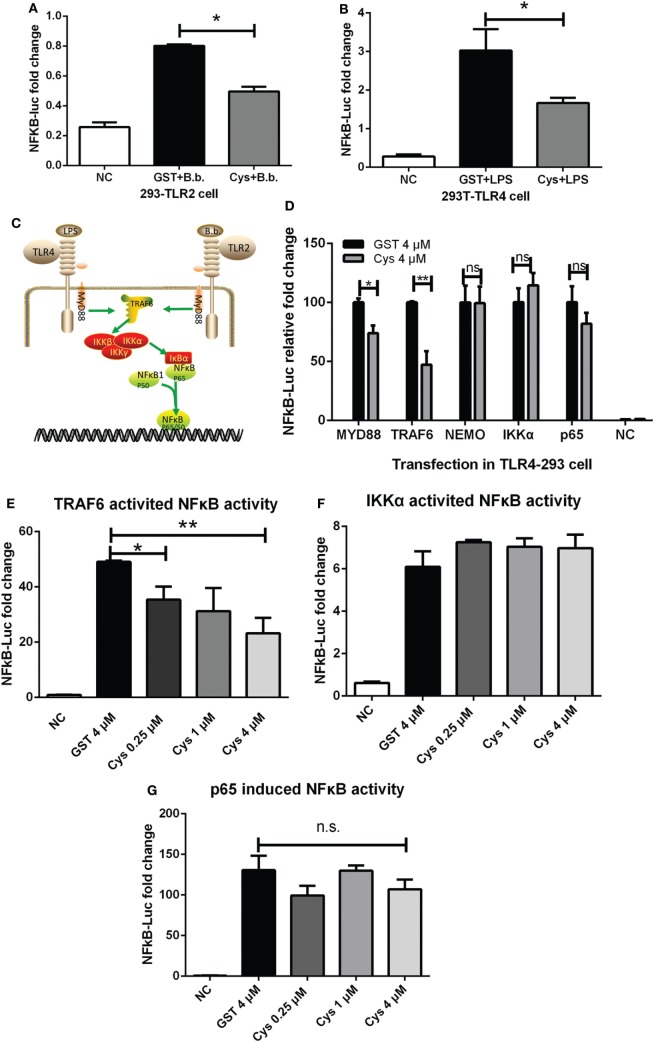
DsCystatin inhibits TLR2 and TLR4-directed NFκB activation. **(A,B)** DsCystatin inhibits *Borrelia burgdorferi*-induced NFκB activation in TLR2-293 cells **(A)** and LPS-induced NFκB activation in TLR4-293 cells **(B)**, as determined by NFκB Luciferase reporter assay. Cells that were not stimulated with LPS or *B. burgdorferi* were served as negative controls (NC). Results are expressed as mean + SEM of three triplicate samples. *p < 0.05 and **p < 0.01 (*t*-test). The representative results from at least three independent experiments are shown. **(C)** A brief diagram of LPS or *B. burgdorferi*-induced TLRs signal pathway. **(D)** DsCystatin inhibits TLR4 signaling pathway at the TRAF6 level. NFκB luciferase reporter was activated by overexpression of distinct signal molecules (MyD88, TRAF6, NEMO, IKKα, and NFκBp65) in TLR4-293 cells. The inhibition of DsCystatin on these molecule-directed NFκB activity was determined by Dual Luciferase reporter assay. **(E)** DsCystatin inhibits TRAF6 overexpression-induced NFκB activity in a dose-dependent manner. **(F,G)** DsCystatin could not inhibit IKKα or NFκBp65 overexpression-induced NFκB activation. Results are expressed as mean + SEM of three triplicate samples. *p < 0.05 and **p < 0.01 (*t*-test). n.s.: not significant. The representative results from at least three independent experiments are shown.

In an effort to reveal the mechanisms, we tested whether signaling pathways leading to induction of pro-inflammatory cytokines are affected by DsCystatin. Gene expressions of inflammatory cytokines are controlled by several transcription factors and one of the key indicators is NFκB. NFκB Luciferase reporter assay suggested that DsCystatin impaired the activation of NFκB in *B. burgdorferi*-stimulated TLR2-293 cells (Figure 3A). Consistent with this, DsCystatin also suppressed LPS-induced NFκB reporter activity in TLR4-293 cells (Figure 3B).

TLR4 signaling pathway is responsible for LPS-induced inflammatory response which activates NFκB. This activation is mediated through MyD88 and TRAF6, followed by subsequent activation of kinases (Figure 3C). We explored how DsCystatin affected the activation of NFκB at the specific points in TLR4 signaling. TLR4-293 cells were pre-incubated with DsCystatin, and NFκB luciferase reporter was activated by overexpression of distinct signal molecules (MyD88, TRAF6, NEMO, IKKα, and NFκBp65) (Figure 3D). NFκB activities induced by overexpression of MyD88 and TRAF6 were significantly inhibited by DsCystatin (Figures 3D,E). However, in the case of overexpression of NEMO, IKKα, and NFκBp65, DsCystatin had no effect on the following activation of NFκB activity (Figures 3D,F,G). So we speculated that DsCystatin may play a role at the level of TRAF6.

### DsCystatin Downregulates TRAF6 Protein Thereby Impairing Nuclear Transport of NFκB

**Figure 4:**
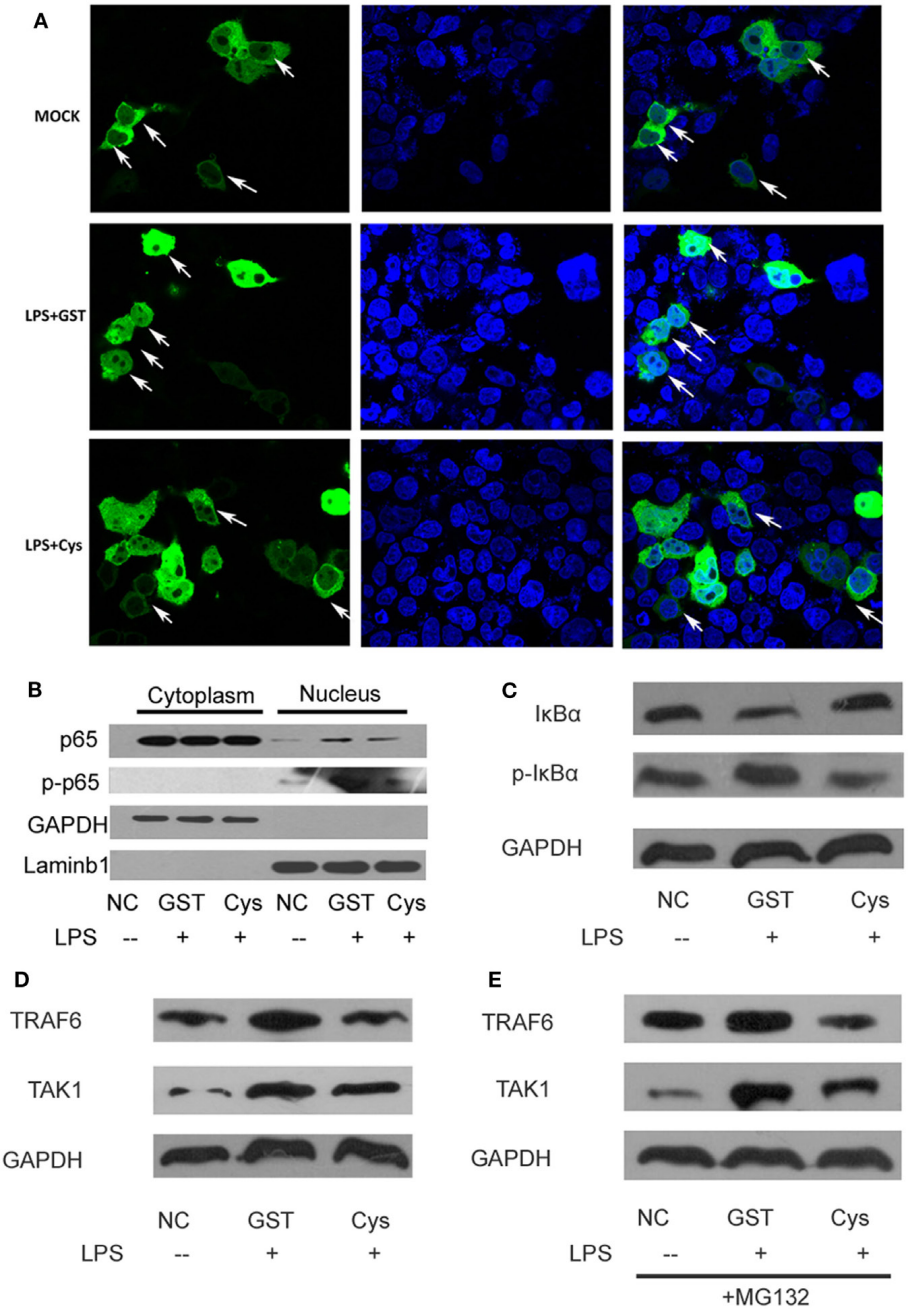
DsCystatin downregulates TRAF6 protein and impairs nuclear transport of NFκB. **(A)** DsCystatin impairs LPS-induced NFκB (p65) nuclear translocation. **(B)** Reduced NFκB p65 protein in nucleus of LPS-stimulated cells incubated with DsCystatin, compared to cells incubated with GST [analyzed by anti-p65 and anti-phosphorylated NFκB p65 (p-p65) antibodies]. **(C)** DsCystatin reduced the phosphorylation and degradation of IκBα during LPS stimulation. **(D)** DsCystatin downregulates the protein level of TRAF6 and TAK1 in LPS-stimulated TLR4-293 cells. **(E)** DsCystatin downregulates TRAF6 and TAK1 level in a proteasome-independent manner. TRAF6 and TAK1 proteins still reduced in DsCystatin-treated cells in the presence of MG132, the typical proteasome inhibitor. The representative results from at least three independent experiments are shown.

Since DsCystatin downregulated LPS and *B. burgdorferi* induced NFκB activity, we further investigated how DsCystatin could influence the expression or activation of proteins involved in the TLR signaling. Immunofluorescence assay suggested that LPS-induced NFκB nuclear translocation was interfered with DsCystatin in TLR4-293 cells (Figure 4A). Consistent with this, we also found a decreased level of phosphorylated NFκB p65 in nucleus after LPS stimulation in DsCystatin-treated cells compared with controls (Figure 4B). The phosphorylation form of IκBα was decreased while the total IκBα was increased in DsCystatin-treated cells (Figure 4C). Increased IκBα protein may account for the blockage of NFκB nuclear translocation during DsCystatin treatment.

The above mentioned experiments suggested that DsCystatin may interfere with TLR signaling at the level of TRAF6. We thereby tested whether DsCystatin influence the protein level of TRAF6 after LPS stimulation. As shown in Figure 4D, TRAF6 protein was elevated upon LPS stimulation in GST-treated control cells, while this increase was significantly weakened in DsCystatin treated cells. TAK1 is the substrate of TRAF6 which can be ubiquitinated by TRAF6 *via* its E3 ligase activity (28). Ubiquitinated TAK1 can be further phosphorylated and then amplify the downstream signaling. Therefore, DsCystatin treatment also downregulated the protein level of TAK1 compared with controls. Interestingly, the downregulation of TRAF6 and TAK1 by DsCystatin was not blocked by MG-132 (Figure 4E), a well-known proteasome inhibitor, which suggested that DsCystatin downregulates LPS-induced TRAF6 expression in a proteasome-independent manner.

### DsCystatin Downregulates TRAF6 Protein *via* an Autophagy-Dependent Manner

**Figure 5:**
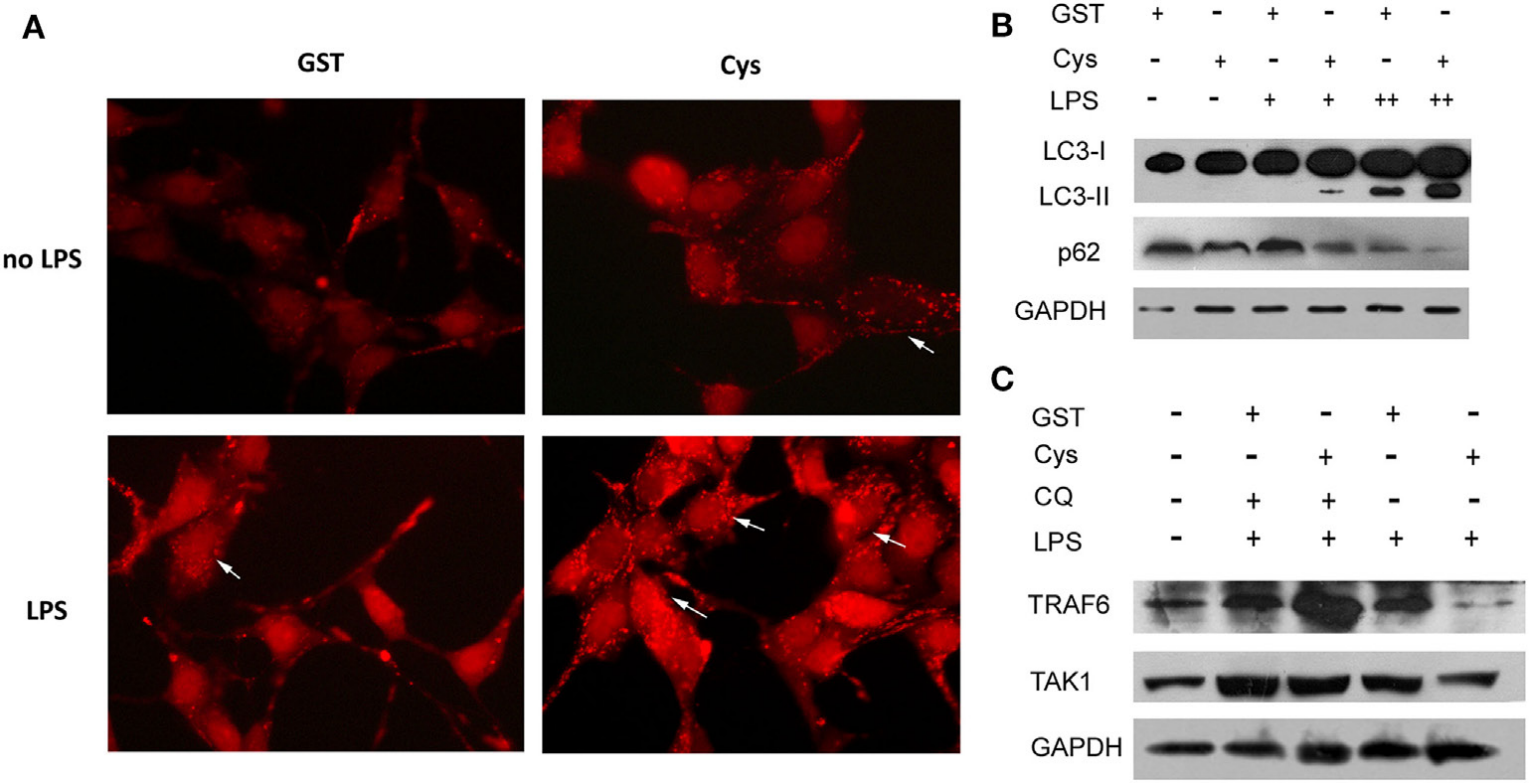
DsCystatin enhances LPS-induced autophagy, thereby leads to the degradation of TRAF6 *via* an autophagy-dependent manner. **(A)** DsCystatin enhances LPS-induced autophagy in TLR4-293 cells. The autophagosome formed by accumulation of mCherry-LC3 was indicated by arrows. **(B)** DsCystatin enhances LPS-induced autophagy in TLR4-293 cells as indicated by Western blotting analysis of autophagy markers LC3-II and p62. **(C)** The degradation of TRAF6 induced by DsCystatin is blocked in the presence of autophagy inhibitor chloroquine (CQ) [LPS + (1 µg/ml), ++ (5 µg/ml)]. The representative results from at least three independent experiments are shown.

By tracking the literature, we have found that TRAF6 can also be degraded *via* autophagy-dependent lysosomal degradation pathway (29, 30). Interestingly, mammalian Cystatin C was reported to induce autophagy in neuron cells to prevent stress-induced cell apoptosis (31, 32). Given that DsCystatin shares a high homology with mammalian Cystatin C (with 44% of sequence similarity to human Cystatin C), we speculated that DsCystatin could enhance autophagy during LPS stimulation. Immunofluorescence assay clearly showed that DsCystatin increased the LPS-induced autophagy as indicated by accumulation of mCherry-LC3 (which forms the autophagosomes) (Figure 5A). Western blots also confirmed that DsCystatin treatments increased LC3-II (autophagosome marker) protein and decreased p62 (a marker for the completion of autophagy) protein (Figure 5B). Most importantly, DsCystatin did not decrease TRAF6 and TAK1 protein expression in the presence of an autophagy inhibitor CQ (Figure 5C). These data suggested that DsCystatin enhances LPS-induced autophagy, which in turn contributes to the degradation of TRAF6 *via* an autophagy-dependent manner.

### DsCystatin Relieves *B. burgdorferi*-Induced Joint Inflammation in C3H/HeJ Mice

**Figure 6:**
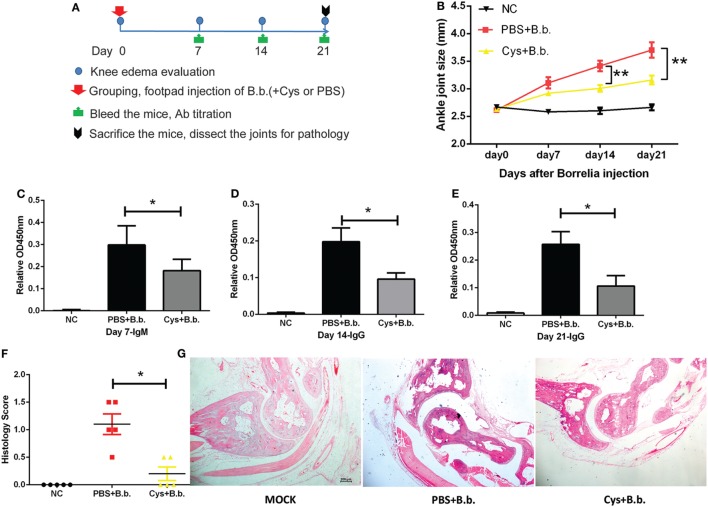
DsCystatin relieves *Borrelia Burgdorferi*-induced joint inflammation in C3H/HeJ mice. **(A)** The protocol of DsCystatin treatment on *B. burgdorferi*-induced joint inflammation. **(B)** DsCystatin reduced the size of ankle joints after *B. burgdorferi* infection compared with control mice. The number of animals per group is five in each experiment. Results are expressed as mean ± SEM of ankle joint thickness, *n* = 5. **p < 0.01 compared to PBS group (multiple *t*-test). The representative results from three independent experiments are shown. **(C–E)** Antibody response decreased in DsCystatin-treated mice compared with controls: **(C)** *B. burgdorferi* specific IgM level at Day 7 post infection. **(D,E)** *B. burgdorferi*-specific IgG level at Day14 **(D)** and Day 21 **(E)** post infection. Results are expressed as mean + SEM value of antibody titers from five mice. *p < 0.05 (*t*-test). The representative results from three independent experiments are shown. **(F)** Arthritis score of *B. burgdorferi*-infected mice treated with DsCystatin or PBS control. Results are expressed as mean ± SEM of arthritis scores of five mice. *p < 0.05 (*t*-test). The representative results from three independent experiments are shown. **(G)** H&E staining of joint sections from *B. burgdorferi*-infected mice at Day 21 post infection. The representative results from three independent experiments are shown.

Since DsCystatin displays anti-inflammatory properties in our *in vitro* study, we further investigate whether DsCystatin has the immunomodulatory activity *in vivo*. We assessed its effect in a mouse model of *B. burgdorferi*-induced joint inflammation in C3H/HeJ mice. 10^3^ *B. burgdorferi* was administered in the mouse footpads in the presence or absence of GST-free DsCystatin (Figure 6A). When *B. burgdorferi* was injected in the presence of DsCystatin, an inhibition of ankle joints swelling was observed (Figure 6B). Next, we analyzed the titers of *B. burgdorferi*-specific antibodies in serum by OspC antigen-based ELISA. As shown in Figures 6C–E, the *B. burgdorferi* OspC-specific IgM (at day 7) and IgG (at day 14 and 21) were significantly decreased in DsCystatin-treated mice when compared with control mice. At 21 days post infection, the mice were sacrificed, and the joint tissues were dissected for pathological analysis. As expected, synovitis and destruction of bone and cartilage were observed in infected control mice; however, these pathological changes were alleviated in DsCystatin-treated mice. In addition, the scores of synoviocytes hyperplasia, cellular infiltration, and cartilages and bone erosion were also reduced in DsCystatin-treated mice compared with those in control mice (Figures 6F,G).

### Role of DsCystatin in the Treatment of CFA-Induced Joint Inflammation

**Figure 7:**
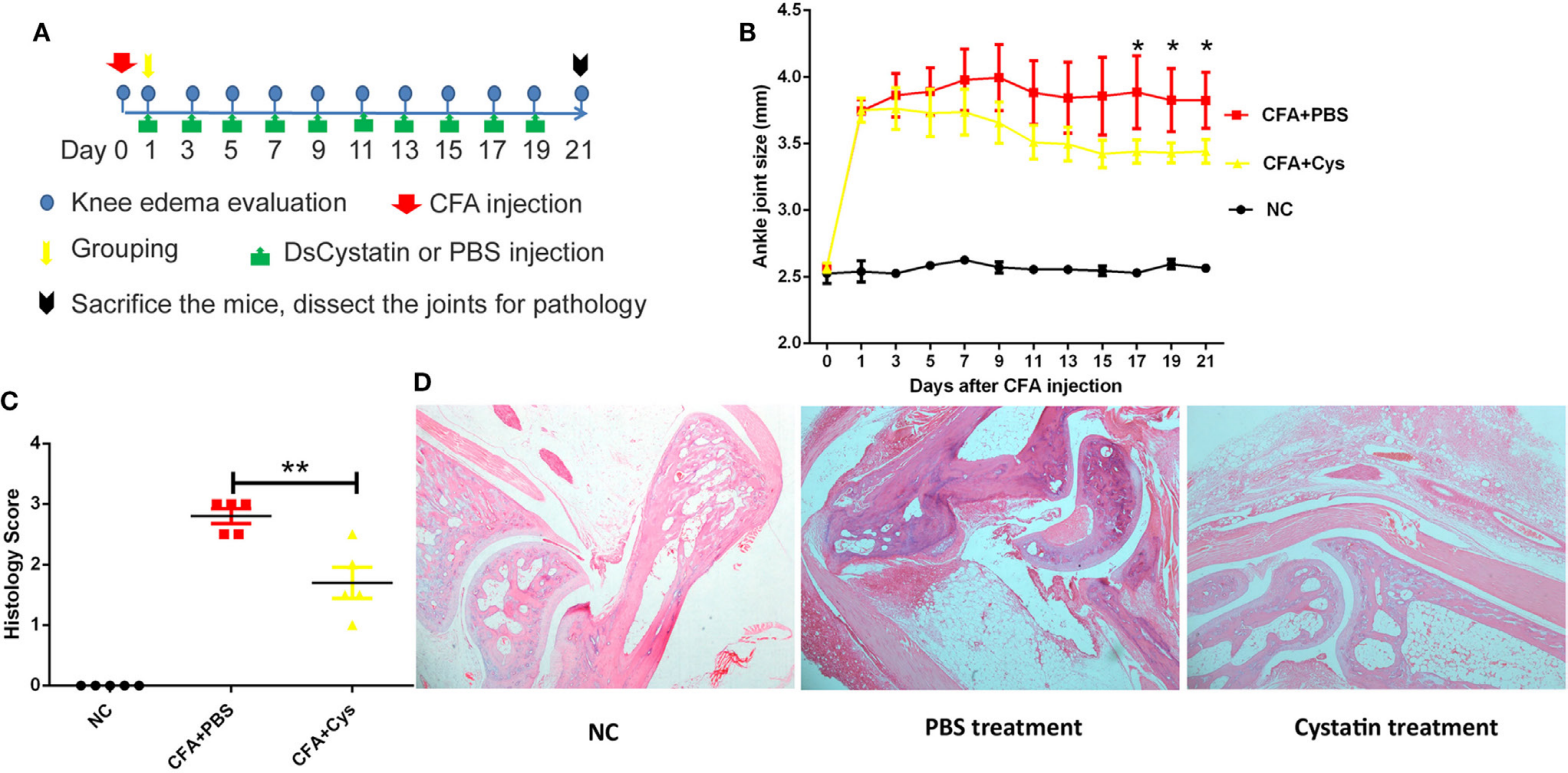
The role of DsCystatin in treatment of complete Freund’s adjuvant (CFA)-induced joint inflammation. **(A)** The protocol of DsCystatin on treatment of CFA-induced joint inflammation. **(B)** DsCystatin treatment reduced the joint swelling after CFA inoculation compared with controls. The number of animals per group is five in each experiment. Results are expressed as mean ± SEM of ankle joint thickness, *n* = 5. *p < 0.05 compared to the PBS group (multiple *t*-test). The representative results from three independent experiments are shown. **(C)** Arthritis score of CFA-injected mice treated with DsCystatin or PBS control. Results are expressed as mean ± SEM of arthritis scores of five mice. **p < 0.01 (*t*-test). The representative results from three independent experiments are shown. **(D)** H&E staining of joint sections from CFA injected mice at Day 21 post infection after DsCystatin or PBS treatments. The representative results from three independent experiments are shown.

Intra-articular injection of CFA into the knee joint results in local inflammation and causes negligible systemic disease (25, 26). The arthritis is characterized by swelling, immune cell infiltration, damage to the joint, and pain (26). Next, we used CFA-induced joint inflammation mouse model to explore whether DsCystatin can prevent CFA-induced mouse ankle swelling. CFA or PBS (NC control) was administered in the mouse articular cavity. One day after, mice received 20 µg of GST-free DsCystatin by intramuscular injection near the joints every 2 days (Figure 7A). A persistent inhibition of ankle joints swelling in DsCystatin-treated mice was observed compared with controls (Figure 7B). Subsequently, mice were sacrificed and the joint organs were dissected for pathological analysis at day 21. Histologic analysis demonstrated that DsCystatin treatment reduced the joint inflammation after CFA inoculation compared with controls. H&E staining revealed increased immune cell infiltration, cartilage destruction, bone erosion, and a hypertrophy of synovial tissue in control mice when compared with DsCystatin-treated mice (Figures 7C,D). These data suggest that DsCystatin could also significantly relieve CFA-induced joint inflammation.

## Discussion

Nowadays, the control of the inflammatory responses during infections or autoimmune diseases is still an important problem in the medical practice. It is of great importance to develop new immunosuppressive agents for the treatment of inflammatory diseases.

NFκB is the key transcription factor for the transcription of pro-inflammatory cytokines. The TLR and IL-1R families require MyD88 to recruit kinase IRAK1, IRAK4, and TRAF6 to activate NFκB (28, 33). Studies have shown that Cystatin family members can regulate NFκB activation in various ways (11, 12). Cystatin B deficiency leads to an increase in caspase-11 gene expression, which further activates NFκB, leading to enhanced IL-1 processing and secretion (34). This inhibition is not associated with lysosomal instability and increased Cathepsin activity in the cytoplasm. Overexpression of Cystatin E/M leads to a decrease in phosphorylation of IKKβ and IKBα, leaving NFκB in the cytoplasm, which in turn leads to a decrease in the expression of cytokines and growth factors (12).

In case of tick Cystatin family, Cystatin from *I. scapularis*, Sialostatin L, inhibits production of IL-9 from mast cell, but does not affect IL-6 expression and mast cell degranulation (17). Sialostatin L inhibits DC migration and maturation, affecting DC antigen delivery to T cells, thereby inhibiting the proliferation of T cells (16, 21, 35). Mechanically, Sialostatin L2 reduces the production of TNFα by inhibiting ERK1/2, NFκB, and PI3K signaling (21). RNAseq analysis suggested that Sialostatin L inhibits the expression of IL-9 by downregulation of the expression of IRF-4 (36). However, in most cases, how tick proteins modulate the immune signaling pathways and interfere with the transcription or translation of specific cytokines remains largely unclear.

In our current study, we reported a novel tick cystatin DsCystatin which inhibits NFκB activation in TLR2 and TLR4 signaling pathways. For the first time, we observed that DsCystatin downregulates the protein expression of TRAF6 and TAK1, thereby inhibiting the phosphorylation of IκBα. DsCystatin may lead to TRAF6 degradation in an autophagy-dependent manner. This new mechanism may help to explain how tick Cystatins interfere with host TLR signaling pathway and regulate the expression of inflammatory cytokines from immune cells.

Finally, DsCystatin can relieve the joint inflammation induced by CFA as well as *B. burgdorferi*. These data suggest that DsCystatin is a novel immunosuppressive protein from arthropod vector and can be potentially used in the treatment of inflammatory diseases.

## Ethics Statement

All animal experiments were performed in accordance with the Guide of National Animal Care and Use committee and the Laboratory Animal Ethical Commission of Soochow University (SYXK2014-0030).

## Author Contributions

TS, QW, JW, and JD designed the projects and prepared the manuscript. TS, FW, WP, and QW performed all the experiments and analyzed the data. All authors read and approved the final manuscript.

## Conflict of Interest Statement

The authors declare that the research was conducted in the absence of any commercial or financial relationships that could be construed as a potential conflict of interest.

## Footnotes

^1^https://blast.ncbi.nlm.nih.gov/Blast.cgi (Accessed: March 06, 2015).

^2^http://www.cbs.dtu.dk/services/SignalP/ (Accessed: March 06, 2015).

## Supplementary Material

Figure S1 DsCystatin influences cytokine production in bone marrow-derived macrophages (BMDMs) upon different stimulations. **(A)** DsCystatin suppressed LPS-induced IL1β mRNA expression in mouse BMDM. (GST and recombinant tick proteins P11, Salp20 and Salp17 were served as controls.) **(B)** DsCystatin dose dependently inhibited LPS-induced IL1β, IL6, and IFNγ mRNA expression in mouse BMDM. **(C)** DsCystatin suppressed TNFα (mRNA and protein) and IL1β (protein) expression in IL1β-treated mouse BMDMs. (Cells were treated with 200 ng/ml of IL1β for 12 h, and then the media were changed. After another 6 h, RNA and cell supernatant were harvested for quantitative real-time PCR and enzyme-linked immunosorbent assay analysis.) **(D)** DsCystatin did not influence TNFα and IL1β expression in TNFα-treated BMDM. Results are expressed as mean ± SEM of three triplicate samples. **p* < 0.05 and ***p* < 0.01 (*t*-test). n.s.: not significant. The representative results from at least three independent experiments are shown.

## Abbreviations

*D. silvarum, Dermacentor silvarum*; *B. burgdorferi, Borrelia burgdorferi*; *E. coli, Escherichia coli*; MG, midgut; SG, salivary glands; BMDMs, bone marrow-derived macrophages; BMDCs, bone marrow-derived dendritic cells; qRT-PCR, quantitative real-time PCR; ELISA, enzyme-linked immunosorbent assay; EAE, experimental autoimmune encephalomyelitis; BSK-H, Barbour–Stoenner–Kelly-H; CQ, chloroquine; CFA, Complete Freund’s adjuvant; IPTG, isopropyl β-d-thiogalactoside; DAPI, 4, 6-diamidino-2-phenylindole.
