# Supplementary figures and images for: An Immunosuppressive Tick Salivary Gland Protein DsCystatin Interferes With Toll-Like Receptor Signaling by Downregulating TRAF6
Source: Front Immunol. 2018 Jun 1;9:1245. doi: 10.3389/fimmu.2018.01245 (PMC5996936; doi:10.3389/fimmu.2018.01245)

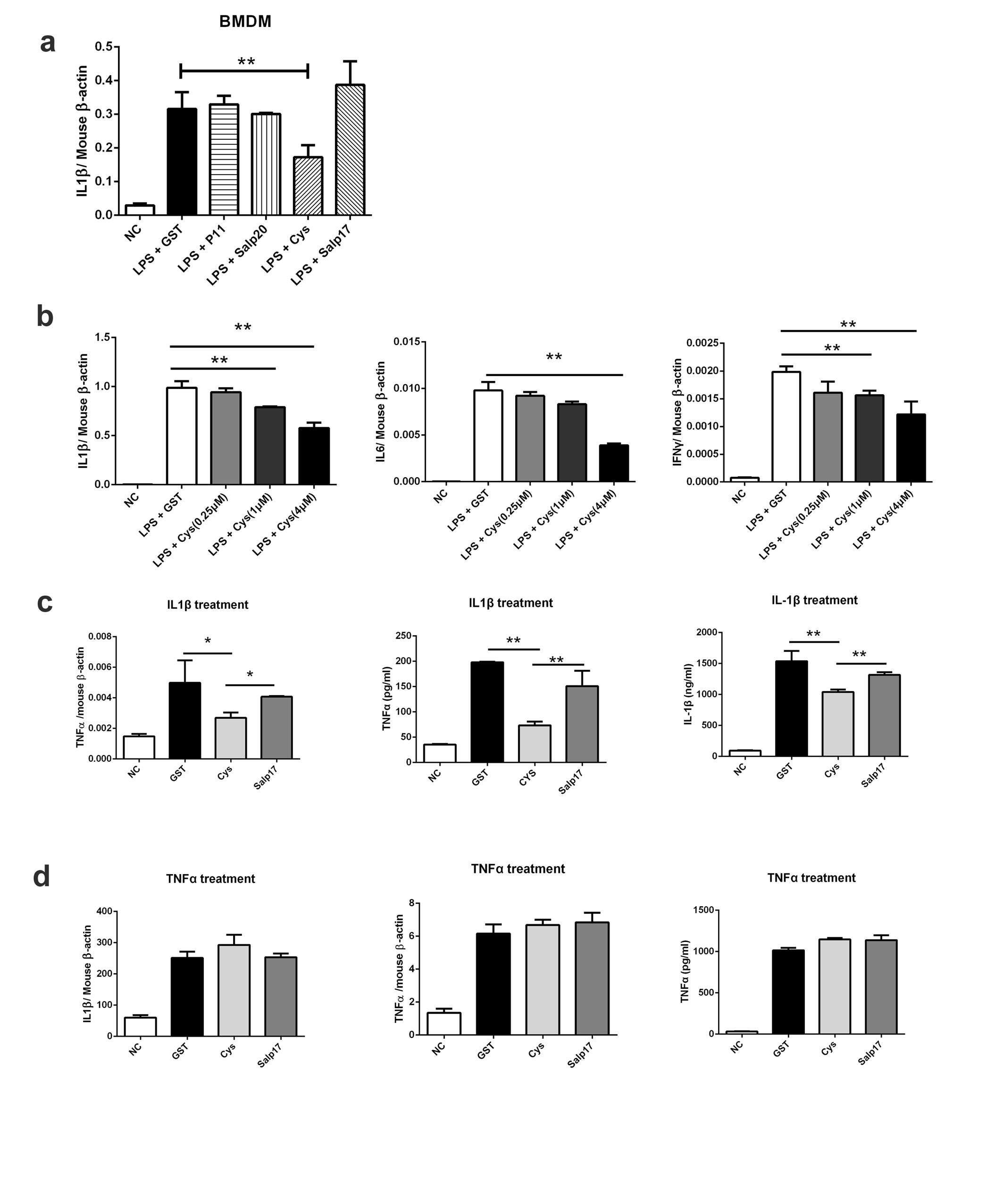

Supplement: Figure S1 — DsCystatin influences cytokine production in bone marrow-derived macrophages (BMDMs) upon different stimulations. (A) DsCystatin suppressed LPS-induced IL1β mRNA expression in mouse BMDM. (GST and recombinant tick proteins P11, Salp20 and Salp17 were served as controls.) (B) DsCystatin dose dependently inhibited LPS-induced IL1β, IL6, and IFNγ mRNA expression in mouse BMDM. (C) DsCystatin suppressed TNFα (mRNA and protein) and IL1β (protein) expression in IL1β-treated mouse BMDMs. (Cells were treated with 200 ng/ml of IL1β for 12 h, and then the media were changed. After another 6 h, RNA and cell supernatant were harvested for quantitative real-time PCR and enzyme-linked immunosorbent assay analysis.) (D) DsCystatin did not influence TNFα and IL1β expression in TNFα-treated BMDM. Results are expressed as mean ± SEM of three triplicate samples. *p < 0.05 and **p < 0.01 (t-test). n.s.: not significant. The representative results from at least three independent experiments are shown. [file Image_1.tif]
