# Supplementary material for: An Immunosuppressive Tick Salivary Gland Protein DsCystatin Interferes With Toll-Like Receptor Signaling by Downregulating TRAF6
Source: Front Immunol. 2018 Jun 1;9:1245. doi: 10.3389/fimmu.2018.01245 (PMC5996936; doi:10.3389/fimmu.2018.01245)
Supplement: Supplementary file 2 [file Table_1.doc]

**Table S1. Sequences of Oligo-primers used in this study.**

| **Target** | **sequence（5’→3’）** |
| --- | --- |
| *Ds Cystatin* | GGAATTCCCATTCCTGGAGGCTGGTCGACG |
|  | CCGCTCGAGAGCGCAGTTGAGGGAAGTGAG |
| *Tick actin* | GCCCTGGACTTCGAGCAGGA |
|  | CAC GTC GCA CTT CAT GAT GG |
| *Mouse β-actin* | AGAGGGAAATCGTGCGTGCGTGAC |
|  | CAATAGTGATGACCTGGCCGT |
| *Mouse TNFα* | ATGGCCTCCCTCTCATCAGT |
|  | ATAGCAAATCGGCTGACGGT |
| *MouseI L1β* | GAGAACCAAGCAACGACAAAATAC |
|  | TTCCCATCTTCTTCTTTGGGTATTG |
| *Mouse IL6* | TTCCATCCAGTTGCCTTCTTGG |
|  | CTTCATGTACTCCAGGTAG |
| *Mouse IFNγ* | AGACAATCAGGCCATCAGCA |
|  | TGGACCTGTGGGTTGTTGAC |
